# Comparative analysis of rabbit hemorrhagic disease virus (RHDV) and new RHDV2 virus antigenicity, using specific virus-like particles

**DOI:** 10.1186/s13567-015-0245-5

**Published:** 2015-09-24

**Authors:** Juan Bárcena, Beatriz Guerra, Iván Angulo, Julia González, Félix Valcárcel, Carlos P. Mata, José R. Castón, Esther Blanco, Alí Alejo

**Affiliations:** Centro de Investigación en Sanidad Animal (INIA-CISA), Valdeolmos, Madrid, Spain; Villamagna SA, Finca “La Garganta”, Villanueva de Córdoba, Córdoba Spain; Department of Structure of Macromolecules, Centro Nacional de Biotecnología/CSIC, Cantoblanco, Madrid, Spain

## Abstract

In 2010 a new Lagovirus related to rabbit haemorrhagic disease virus (RHDV) emerged in France and has since rapidly spread throughout domestic and wild rabbit populations of several European countries. The new virus, termed RHDV2, exhibits distinctive genetic, antigenic and pathogenic features. Notably, RHDV2 kills rabbits previously vaccinated with RHDV vaccines. Here we report for the first time the generation and characterization of RHDV2-specific virus-like particles (VLPs). Our results further confirmed the differential antigenic properties exhibited by RHDV and RHDV2, highlighting the need of using RHDV2-specific diagnostic assays to monitor the spread of this new virus.

## Introduction, methods and results

Rabbit haemorrhagic disease (RHD) is a highly infectious and fatal disease of the European rabbit (*Oryctolagus cuniculus*), (reviewed in [1]). The etiological agent, rabbit haemorrhagic disease virus (RHDV), belongs to the *Lagovirus* genus within the *Caliciviridae* family. This genus comprises several non-pathogenic rabbit caliciviruses, which are genetically related to but relatively distant from RHDV [2,3], and European brown hare syndrome virus (EBHSV). RHDV is highly contagious and usually fatal in adult rabbits, with a mortality range up to 80-100% [4]. Susceptibility to the disease begins in the 5-6^th^ week of life of rabbits and steadily increases up to the 8-9^th^ week when they become fully susceptible. After it was reported in China in 1984, RHD spread rapidly around the world, being currently enzootic in wild rabbit populations in Europe, Australia and New Zealand. When it emerged, RHD dramatically reduced wild rabbit populations and was responsible for great economic losses in the rabbit industry worldwide [1]. Subsequently, efficient inactivated commercial vaccines against RHDV were introduced in the early 1990s, providing a good coverage, since all circulating strains are classified within a single serotype. These vaccines enabled the control of RHD in rabbitries for the last 20 years. Likewise, ELISA methods developed in Italy for the veterinary diagnosis of RHD in domestic rabbits [4,5], were proven effective tools for monitoring RHDV field epidemiology, particularly in surveys conducted in Australia among wild rabbit populations [6,7].

RHDV is a non-enveloped icosahedral single-stranded positive-sense RNA virus. The virus capsid (~40 nm diameter) comprises 90 dimers of a single capsid subunit, the VP60 protein. RHDV as most caliciviruses cannot be grown in cell culture, a fact that has hampered the study of this group of viruses, as well as the development of control measures. A major breakthrough was the finding that expression of recombinant VP60 protein in insect cells results in the formation of virus-like particles (VLPs) that are morphologically and antigenically identical to infectious RHDV virions [8]. RHDV VLPs have been shown to induce full protection of rabbits against a lethal challenge with RHDV [9,10]. These VLPs have also been used for the development of sensitive and reliable tests for detection of antibodies to RHDV [9,11].

The VP60 protein has three domains [12], an N-terminal arm (NTA), a shell (S) forming a scaffold which protects the viral RNA, and a flexible protruding domain (P) at the capsid surface, which contains determinants for virus-host receptor interactions and antigenic diversity [12,13]. The P domain can be further divided into P1 and P2 subdomains, with P2 subdomain located at the outermost surface-exposed region of the viral capsid.

In 2010 a new RHDV related virus with a distinctive pathogenic profile was identified in France [14,15] and has since rapidly spread throughout domestic and wild rabbit populations of Italy, Spain, Portugal Germany, United Kingdom and the Azores islands [14,16-21]. Unlike RHDV, the new lagovirus, termed RHDV2 or RHDVb in the literature, kills rabbit kits under 30 days of age, as well as rabbits that had been previously vaccinated against RHDV. Further studies have pointed out other distinctive features of RHDV2: it causes an average mortality of 20% in experimentally infected rabbits [14], which is consistently less than RHDV, and exhibits a broader host range, since it infects other lagomorphs like different hare species [17,22], causing an RHD-like disease. RHDV2 has specific genetic and antigenic profiles. It has been shown that several monoclonal antibodies (MAbs) against RHDV capsid protein fail to react with cognate RHDV2 virions [14,17].

The situation originated by the emergence of RHDV2 virus has raised concerns regarding the impact of the new disease among domestic and wild rabbit populations [23]. Given the serious epizootic situation created, new inactivated vaccines against RHDV2 have been urgently developed and their use has been provisionally allowed in European Union member states. Recently, the World Organization for Animal Health (OIE) Reference Laboratory for RHD (IZSLER, Brescia, Italy), has developed a serological assay based on the use of specific anti-RHDV2 MAbs, rabbit immune serum and virus capsid antigen obtained from RHDV2-infected rabbit liver extracts [22].

Here we report for the first time the generation and characterisation of RHDV2-specific VLPs, and their use together with RHDV-specific VLPs, to conduct a comparative serological study involving sera from vaccinated and virus-infected rabbits. Our results further confirmed the differential antigenic properties exhibited by RHDV and RHDV2 virus capsids, highlighting the need for using specific serological assays as tools to monitor the epidemiology of this emergent virus, where virus-specific VLPs could represent a convenient alternative to classical virus antigen.

**Figure 1:**
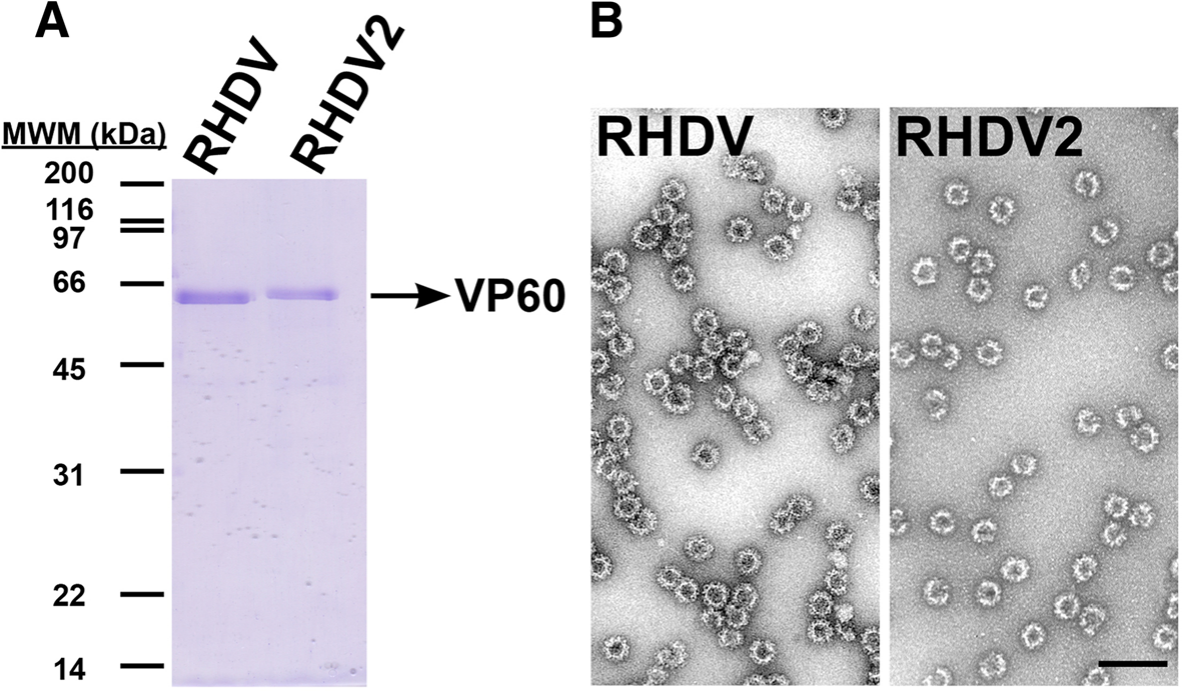
**The RHDV2 recombinant capsid protein assembles into VLPs. A** Coomassie blue stained SDS-PAGE of purified recombinant RHDV and RHDV2 VP60 proteins. Molecular weight markers (MWM) are given on the left (×10^3^ Da). **B** Electron micrographs of negatively stained samples of RHDV and RHDV2 purified VP60 particles. Scale bar, 100 nm.

We generated a recombinant baculovirus expressing the RHDV2 VP60 protein. For comparative purposes, a baculovirus expressing RHDV VP60 was generated in parallel. Codon-optimized genes for expression in insect cells of the RHDV (strain Ast89, GenBank accession no. Z49271) and RHDV2 (strain Vs11, GenBank accession no. JX106023) VP60 proteins were synthesised (GenScript, USA) and subcloned into BglII/EcoRI digested plasmid pHAPh2GS [24]. Recombinant baculoviruses were obtained by cotransfection with the flashBACULTRA linearized baculoviral genome (Oxford expression technologies, UK) as indicated by the manufacturer. Both VP60 proteins were expressed to grossly similar levels in infected H5 insect cells (not shown). Subsequently, in order to determine whether the VP60 constructs were able to assemble into particulate material, infected-cell extracts were subjected to a VLP-purification protocol as previously described [25]. As shown in Figure 1A, both VP60-derived assemblies were purified to near homogeneity. Electron microscopy analysis of negatively stained preparations revealed that both, RHDV and RHDV2-derived VP60 proteins assembled with similar efficiency into VLPs of approximately 40 nm in diameter (Figure 1B), which were morphologically identical to previously described RHDV VLPs [8,26].

As a first step in the assessment of the antigenic profile of both lagovirus VLPs, we evaluated their reactivity with a panel of three MAbs, raised against RHDV VLPs in our laboratory following previously described procedures [27]. We also included in this analysis the capsid protein of EBHSV, expressed in the baculovirus expression system, available in our laboratory. The MAbs had been previously characterized by assessing their reactivity with a set of RHDV VP60 deletion mutants, as well as a set of overlapping peptides encompassing the complete P2 subdomain (unpublished results). The analysis was performed using an indirect ELISA assay. Briefly, purified VLPs (0.5 μg/mL in PBS buffer) were absorbed to Nunc Polysorp ELISA plates. Next, serial three-fold dilutions of the MAbs (hybridoma supernatants) were added to the wells, followed by addition of HRP-conjugated goat anti-mouse IgG (H + L) (Invitrogen) at a 1/3000 dilution. Table 1 summarizes the results obtained. MAb 2E7, which recognizes a linear epitope located at the C-terminal end of VP60 protein (within aa positions 549-579), at the P1 subdomain, reacted with the three lagovirus capsid proteins tested. MAb 1G5, which recognizes a linear epitope with the sequence: NPISQVAP (aa positions 326-333 of VP60 protein) located at loop L2 of P2 subdomain, reacted with both, RHDV and RHDV2 capsid proteins, but not with the cognate EBHSV capsid protein. Finally, MAb 1C9 reacted only with the RHDV capsid protein. This MAb binds to a conformational epitope, as it does not react with RHDV capsid protein in Western blot, or in ELISA assays where the VP60 protein was denatured by treatment with 7 M urea before adsorption. Interestingly, MAb 1C9 does not bind to modified RHDV VLPs, derived from VP60 deletion or insertion mutants, lacking the amino acid sequence PGNNAT, which corresponds to aa positions 305-310, and is located within loop L1 at the tip of the P2 subdomain of the VP60 protein. These results were consistent with the available information regarding sequence alignment studies of lagovirus capsid proteins [3,14,28], which indicate the regions recognized by MAbs 2E7 and 1G5 are relatively conserved among the viruses tested, while the sequence corresponding to aa 305-310, critical for MAb 1C9 binding, lies within variation region V1 [12,14], one of the seven highly variable regions defined on VP60 capsid protein, which has been shown to constitute a major neutralization site [12]. Altogether, these data suggests MAb 1C9 targets an antigenically relevant epitope on the RHDV capsid, not present in RHDV2, which deserves further characterization.

**Table 1:** **Reactivity of anti-RHDV MAbs against the capsid proteins of RHDV, RHDV2 and EBHSV in indirect ELISA assays.**

MAb	RHDV	RHDV2	EBHSV	Epitope location (aa position within VP60 protein)
2E7	+	+	+	(aa 549-579, P1 subdomain)
1G5	+	+	-	NPISQVAP (aa 326-333, loop 2 at P2 subdomain)
1C9^a^	+	-	-	PGNNAT (aa 305-310, loop 1 at P2 subdomain)

+ Positive reaction; - Negative reaction.

^a^ 1C9 is a conformational epitope. It does not bind to RHDV VLPs derived from VP60 deletion or insertion mutants lacking the sequence corresponding to aa positions 305-310 of VP60 protein.

**Figure 2:**
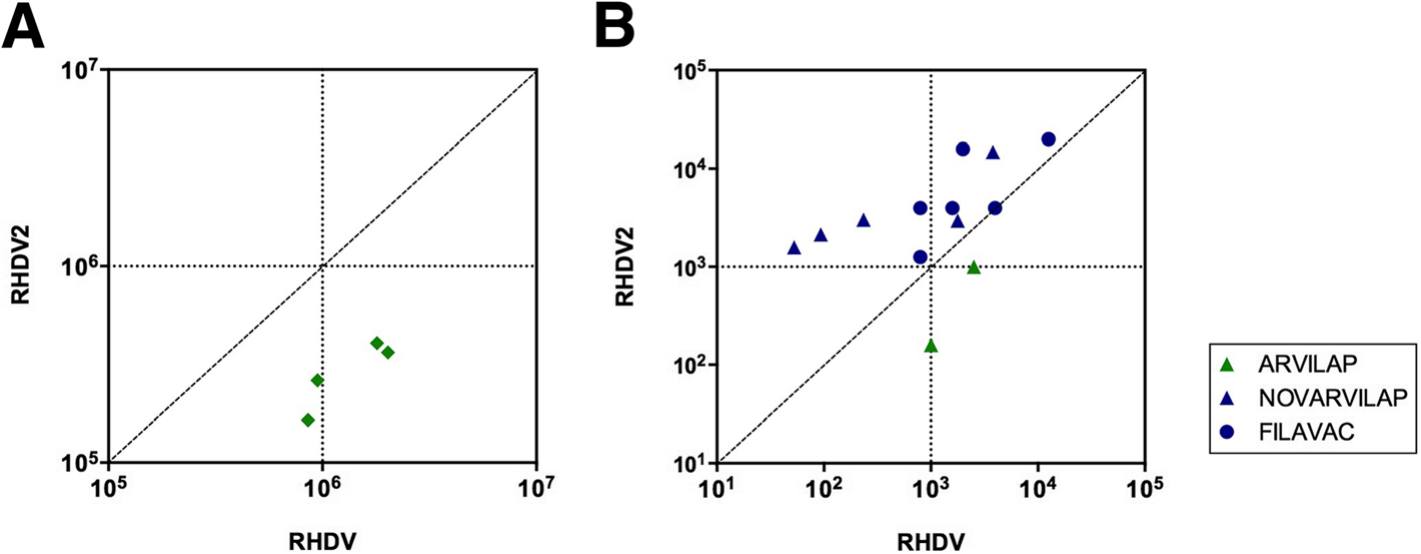
**Antibody responses in RHDV and RHDV2 vaccinated rabbits analyzed by indirect ELISA. A** Titres of sera (*n* = 4) from rabbits immunized with RHDV VLPs. Each sample is represented on a coordinate graph showing its titre for RHDV on the x-axis and for RHDV2 on the y-axis. For the determination of serum titres, seven serial three-fold dilutions starting from 1/10 000 were tested by ELISA for reactivity against RHDV and RHDV2 VLPs independently in duplicate samples. OD measurements were blank-corrected and titre extrapolated as the dilution at which they reached an arbitrary OD value (0.240) corresponding to that obtained by the mean + 2 times the standard deviation of 12 serum samples from non-vaccinated animals assayed at a 1/30 dilution. This OD value was initially established as threshold to classify each serum as negative or positive. All calculations were performed using GraphPad software. **B** Titres of sera from 8 week old rabbits vaccinated with ARVILAP (*n* = 2) or 4 week old rabbits vaccinated with FILAVAC (*n* = 5) or NOVARVILAP (*n* = 5), respectively. ELISA assays were performed as described above using a dilution range starting from 1/30. Representation and calculations are as in panel A. Procedures involving obtainment of animal samples were carried out under EU guidelines and supervised and approved by the institutional ethical review committee.

We then assessed whether polyclonal rabbit sera might also recognize RHDV and RHDV2 capsids differently. To this end, rabbit hyperimmune sera raised against RHDV VLPs were tested by indirect ELISA against both RHDV and RHDV2 VLPs. Briefly, RHDV or RHDV2 VLPs (0.5 μg/mL in PBS buffer) were absorbed to Nunc Polysorp ELISA plates. Next, sera obtained from rabbits inoculated four times with 500 μg of RHDV VLPs emulsified in Montanide ISA 50 V2 (Seppic, France), were assayed using seven serial three-fold dilutions starting from 1/10 000. Subsequently, the secondary antibody (goat anti-rabbit IgG (H + L) HRP-conjugate; Thermo Fisher Scientific) was used at 1/3000 dilution, followed by addition of substrate solution (OPD (*o*-phenylenediamine dihydrochloride); Invitrogen). The reaction was stopped by addition of 3 N H_2_SO_4_, and color development was recorded at 492 nm. In Figure 2A, sera titres are represented as dots with their x-axis value corresponding to the titre obtained against RHDV VLPs and the y-axis value to the titre obtained against RHDV2 VLPs. All sera tested showed higher antibody titres against RHDV VLPs, their cognate antigen, than against RHDV2 VLPs, as indicated by the fact that the titre levels obtained were all situated below the diagonal line represented on the graph.

Using the same approach (with a dilution range starting from 1/30), we tested sera from rabbits vaccinated against RHDV (ARVILAP vaccine, Laboratorios Ovejero, Spain) or RHDV2 (NOVARVILAP vaccine, Laboratorios Ovejero, Spain or FILAVAC VHD VARIANT vaccine, Filavie, France) collected 21 days after vaccination (Figure 2B). All animals developed specific antibody responses which were consistently higher for the cognate than for the heterologous antigen. Taken together, the results obtained revealed a markedly different antigenicity between both viral capsids, which is consistent with the observed lack of efficient protection afforded by current commercial RHDV vaccines against RHDV2.

**Figure 3:**
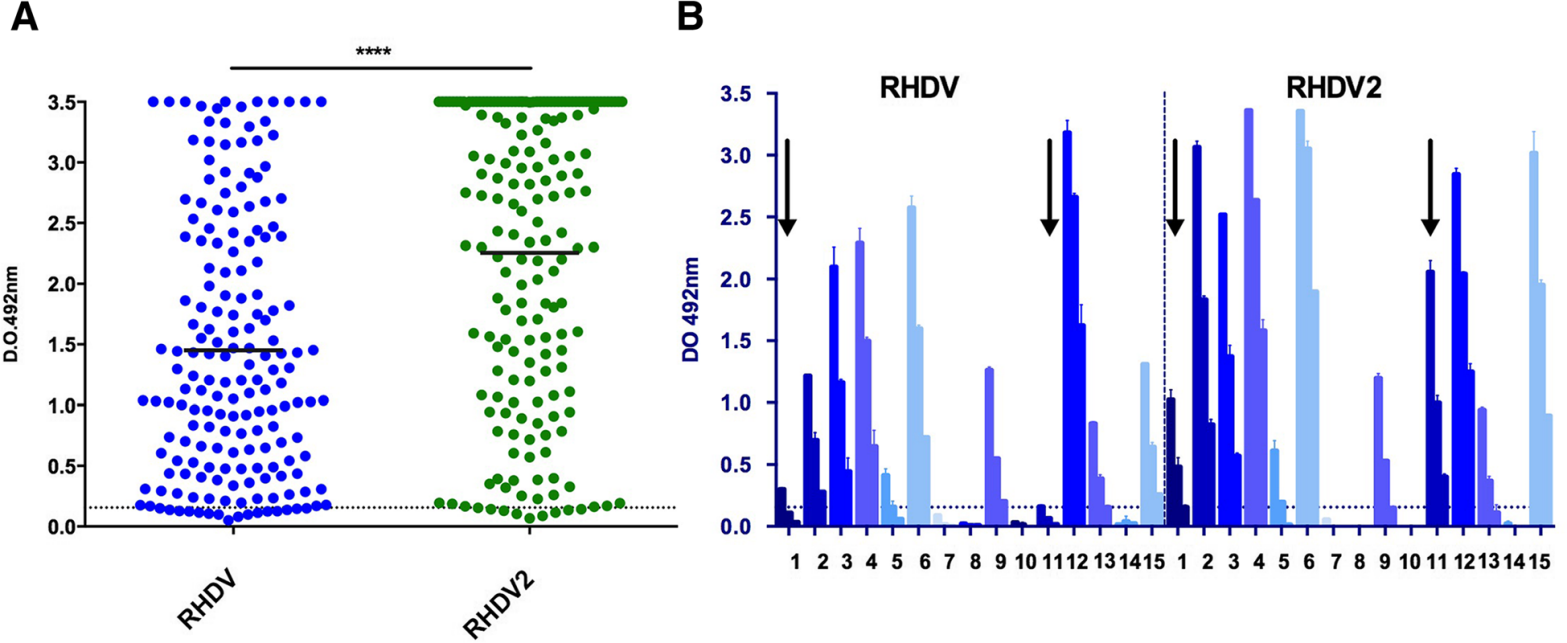
**Prevalence of anti-RHDV and anti-RHDV2 antibodies in a wild rabbit population analyzed by indirect ELISA. A** A collection of 180 sera from wild rabbits captured in a region of Central Spain (Ciudad Real) during the months of April and November 2014 was tested by ELISA using plates coated with RHDV or RHDV2 VLPs. A single dilution (1/500) of each sample was tested and recorded OD values for each sample are represented as two separate groups. A line at an arbitrary value (0.155) corresponding to the mean OD+ 2 times the standard deviation of 10 samples from non vaccinated animals at the same dilution is shown. Mean plus SD are indicated for each antigen group. Asterisks indicate a statistically significant difference (*p* < 0.0001) as assessed by a paired t-test. **B** Representation of OD values obtained using three different dilutions (1/300, 1/900 and 1/2700) of 15 selected sera from the same collection. ELISAs were performed in duplicate under the same conditions indicated above. Arrows highlight sera samples showing reactivity with RHDV2 but not with RHDV VLPs. Procedures involving obtainment of animal samples were carried out under EU guidelines and supervised and approved by the institutional ethical review committee.

Finally, we used both RHDV and RHDV2 VLPs to screen by ELISA a set of 180 serum samples collected from a wild rabbit population during April-November 2014, in a region of Central Spain (Ciudad Real). Figure 3A shows the results obtained when a single dilution (1/500) of each serum sample was tested. A high prevalence of antibodies to lagoviruses was detected among the apparently healthy rabbit population analyzed, with more than 91% of the sera samples reacting with RHDV VLPs and 95% with RHDV2 VLPs. The mean signal obtained was significantly higher when RHDV2 VLPs were used as antigen, a difference which was most likely an underestimate, given the large fraction of saturated samples in this assay. Importantly, 7 serum samples were shown to react with RHDV2 but not with RHDV-VLPs under the ELISA conditions used. A more detailed analysis (three different dilutions of each sample: 1/300, 1/900 and 1/2700) was performed with 15 randomly selected sera samples (Figure 3B). Of these, four showed no detectable signal at the tested dilutions. Sera from two animals reacted with RHDV2 VLPs but not with RHDV VLPs, while of the remaining nine, five showed higher signal to RHDV2 than to RHDV and four had comparable reactivity. None of the analysed sera showed higher titres towards RHDV than to RHDV2. Overall, the results obtained suggested that the novel virus RHDV2 had circulated among the studied wild rabbit population, being responsible for at least great part if not all of the observed seroconversion.

## Discussion

The available information regarding sequence alignment studies of lagovirus full-length capsid proteins indicate RHDV2 is phylogenetically distinct from all previously described members of the genus *Lagovirus* and forms a new genetic group [14]. Amino acid identity among RHDV2 strains is close to 97%, whereas average identity is 89.2% when compared with RHDV strains [14,17]. When the sequence comparison is restricted to the 7 regions of the P domain that show the highest degree of genetic variation (regions V1 to V7 [12]), the identity between RHDV2 and RHDV strains drops to 60% [14]. Moreover, the crystallographic resolution of the VP60 protruding P domains of RHDV [12] and RHDV2 [29], has enabled the analysis of structural differences between both viruses. Although their overall structures are similar, the P1 subdomain helices are slightly shifted and some P2 subdomain loops are oriented differently. Interestingly, amino acid changes between both viral capsid proteins tend to concentrate on the three extended loop regions located at the outer surface of the P2 subdomain [29]. These results provide insights regarding the marked antigenic differences observed between both viruses, remarkably the lack of efficient protection against RHDV2 afforded by current RHDV inactivated vaccines, which has prompted the urgent development and provisional authorization of RHDV2-specific inactivated vaccines.

Epidemiological surveillance of RHD among domestic and wild rabbit populations is performed using serological assays based on the use of RHDV antigen obtained from infected-rabbit liver extracts and virus specific MAbs [4-6]. Commercial test kits based on RHDV VLPs are currently available and have been used in several studies [16,30]. In Australia, early seroepidemiologic surveys [7] evidenced the circulation of a non-pathogenic rabbit calicivirus (RCV-A1), which was subsequently identified and characterized [2]. Serological assays enabling discrimination of both viruses, based on the use of virus specific VLPs, have been developed [11]. In Europe, the rapid spread of the newly emergent virus RHDV2, which is apparently replacing classical RHDV strains among rabbit populations [14,18] and is capable of infecting lagomorphs from the *Lepus* genus [17,22], is a major cause of concern. The availability of virus specific reagents such as RHDV2 VLPs, would greatly ease monitorization of field evolution and circulation of RHDV2, as well as its interaction with RHDV when both viruses co-circulate in rabbit populations.

Here we report for the first time the generation and characterization of RHDV2-specific VLPs. Our results further confirmed the differential antigenic properties exhibited by RHDV and RHDV2 capsid proteins. Using sera from both, vaccinated and infected rabbits, it was shown that antibody responses were consistently higher for the cognate than for the heterologous antigen. Interestingly, the results obtained in the serological analysis of sera collected from an apparently healthy wild rabbit population from Central Spain, suggesting high prevalence of RHDV2, were in agreement with previous reports indicating rapid spread of the novel virus through the Iberian Peninsula [16,23].

The VLPs reported in this study might also be useful for the development of vaccines against RHDV2. Current provisionally authorised vaccines for RHDV2 are prepared from liver extracts of experimentally infected rabbits. Since RHDV2 induces consistently low mortality rates (20-30%) as compared to RHDV (close to 100%), this might represent an important drawback for vaccine production (i.e. lower yields of virus antigen per infected rabbit). Therefore, the development of new recombinant subunit vaccines for RHDV2 based on VLPs might provide greater benefit than the equivalent for RHDV.

In addition, this work enabled the characterization of our MAbs 2E7, 1G5 and 1C9, regarding their differential reactivity with three lagovirus capsid proteins (RHDV, RHDV2 and EBHSV). In summary, the RHDV2 VLPs and the MAbs reported in this study provide tools that might be useful for monitorization of virus circulation, development of control measures, as well as for research in different aspects of the biology of this relevant emergent virus.
